# Patient with severe fever with thrombocytopenia syndrome virus infection and central nervous system disturbance in Dongyang, Zhejiang Province, China, 2017

**DOI:** 10.1186/s12985-019-1230-9

**Published:** 2019-11-07

**Authors:** Yi Sun, Bin Guo, Hao Yan, Ai Lan Wu, Wen Wu Yao, Kang Chen, Jun Hang Pan, Zhao Xia Li, Hai Yan Mao, Yan Jun Zhang

**Affiliations:** 1 Zhejiang Provincial Center for Disease Control and Prevention, 3399 Binsheng Road, Hangzhou, 310051 Zhejiang China; 2 Dongyang Center for Disease Control and Prevention, Dongyang, Zhejiang China; 3 Dongyang People’s Hospital, Dongyang, Zhejiang China

**Keywords:** Severe fever with thrombocytopenia syndrome virus, Central nervous system disturbance, Clinical characteristic, Epidemiological feature, Phylogenetic

## Abstract

**Background:**

Severe fever with thrombocytopenia syndrome (SFTS) is an emerging hemorrhagic fever that was first described in China in 2011. We report a patient who died of *Severe fever with thrombocytopenia syndrome virus* (SFTSV) infection, with a rapidly progressive central nervous system (CNS) disturbance, in Dongyang, Zhejiang Province, China, in 2017.

**Case presentation:**

A 64-year-old man was admitted to hospital after 4 days of fever. SFTSV was detected 1 day after the patient was admitted to hospital. The patient presented with CNS disturbance and died 4 days after admission. Detailed clinical and epidemiological investigations and laboratory tests were conducted. Reduced platelet, white blood cell, lymphocyte, and neutrophil counts, elevated lactate dehydrogenase, creatine kinase, aspartate aminotransferaseand alanine aminotransferase concentrations, and an increased activated partial thromboplastin time were observed. In a phylogenetic analysis, the isolate clustered close to a strain derived from South Korea.

Conclusions: This is the first case of SFTSV infection with CNS disturbance in Dongyang, Zhejiang Province, China. The surveillance of suspected cases of SFTS is important in SFTSV endemic regions.

## Background

*Severe fever with thrombocytopenia syndrome virus* (SFTSV) poses serious public health concerns globally because it causes tick-borne hemorrhagic fever with a high case fatality rate (12–50%) [1–3]. Many cases of SFTSV infection have been confirmed in Zhejiang Province, China, since its first comprehensive description in 2011 [1, 4]. Fever, thrombocytopenia, leukocytopenia, and multi-organ dysfunction have been reported in SFTSV-infected patients. Several studies have reported patients who presented with rapidly progressive disturbances of the central nervous system (CNS), such as a human-encephalitis-like syndrome [5–7]. Here, we report a random case of SFTSV infection presenting with human-encephalitis-like syndrome in 2017. To the best of our knowledge, this is the first case of SFTS with CNS involvement reported in Zhejiang Province, China. Our objectives were to understand (1) the clinical human-encephalitis-like syndrome and the epidemiological and virological characteristics of this case; (2) the importance of the surveillance of suspected cases of SFTS in SFTSV endemic regions.

The SFTS diagnoses were confirmed based on previously described criteria [4]. Clinically diagnosed encephalitis was defined as a condition meeting the following criteria: (a) sudden onset; (b) symptoms of fever, headache, vomiting, etc.; and (c) disorders of consciousness, which have been previously described [6, 8]. Serum samples were collected with the permission of the patient and his wife. Blood samples were collected continuously from the patient during his hospitalization for hematological and biochemical examination, to closely monitor the clinical progression of the disease. The samples were stored in 3–5 mL of Hanks solution containing 100 U/mL penicillin and 100 μg/mL streptomycin, at− 70 °Cuntil analysis. With the permission of the local government, one tick sample was collected from a hill that the patient had visited, using the flagging and dragging method. The tick was delivered to the local Center for Disease Control and Prevention (CDC) and tested for SFTSV.

Viral RNA was extracted with the RNeasy Mini Kit (Qiagen, Redwood City, CA, USA), according to the manufacturer’s instructions. Multiplex Real-time RT–PCR reactions and sequencing of SFTSV were performed as described previously [9]. We constructed multiple alignments with Geneious 11.1.5 (www.geneious.com) using data matrices of SFTSV sequences downloaded from GenBank, according to previous studies [10–12]. Dataset-specific models were selected with the Akaike information criterion in Modeltest 3.7 [13]. A phylogenetic analysis was performed with the general time reversible model as the model of nucleotide substitution and the maximum likelihood (ML) method, and phylogenetic trees based on the different viral segments were constructed with MEGA 7.0.14 (http://www.megasoftware.net/). Segments from Uukuniemi virus (GenBank accession numbers: L, D10759; M, NC_005220; S, NC_005221) were used as outgroups in each segment tree correspondingly. The statistical significance of the constructed phylogenies was estimated with a bootstrap analysis with 1000 pseudoreplicate datasets. The viral sequences from this patient (CMX/Zhejiang/2017) were deposited in GenBank (MK424388–MK424390). Homologous mosaic structures were detected with the Recombination Detection Program v3.29 [14].

## Case presentation

On December 8, 2017, a 64-year-old male retired country doctor who lived in a hilly rural area in Dongyang, Zhejiang Province, China, developed chills, fever, headache, malaise, muscular soreness, nausea, and subconjunctival hemorrhage. He had been healthy up to that point, with no significant underlying illness. He went to a health clinic in town with a temperature of 38.5 °C on December 9 and stayed at home to rest on December 10. He remained symptomatic and was transferred to Dongyang People’s Hospital on the morning of December 11. The patient was recorded as vomiting once and had a fever of 38.5 °C. His platelet (PLT) count was 82 × 10^9^/L, white blood cell (WBC) count was 1.9 × 10^9^/L, neutrophil-granulocyte count was 1.22 × 10^9^/L, and lymphocyte count was 0.53 × 10^9^/L (Table 1). He was admitted to hospital at noon on December 11 with a fever of 38.0 °C. His gingiva was bleeding. A rash was present all over his lower abdomen, waist, and groin area. He was diagnosed with suspected SFTS based on his symptoms. SFTSV infection was confirmed by the local CDC with a positive SFTSV RNA test on the afternoon of December 12. The patient’s PLT count was 76 × 10^9^/L, WBC count was 1.13 × 10^9^/L, neutrophil-granulocyte count was 0.81 × 10^9^/L, and lymphocyte count was 0.3 × 10^9^/L on December 12 (Table 1). Laboratory tests showed elevated lactate dehydrogenase (LDH; 466 U/L), creatine kinase (CK; 580 U/L), alanine aminotransferase (ALT; 54 U/L), and aspartate aminotransferase (AST; 137 U/L) (Table 1). His activated partial thromboplastin time (aPTT; 45.6 s) was longer than normal (Table 1). The patient received the following treatments: (a) both PLTs and white cells were supplied; (b) immunoglobulin and thymosin were used as immune support; and (c) glycyrrhizin was used as an hepatoprotective agent. The patient’s speech was slurred and his condition worsened, with progressive disturbance of consciousness from December 13. His PLT count was 82 × 10^9^/L, WBC count was 2.02 × 10^9^/L, neutrophil-granulocyte count was 1.56 × 10^9^/L, and lymphocyte count was 0.36 × 10^9^/L on December 13 (Table 1). Significant disorder of consciousness was apparent on the morning of December 14, with suddenly rigidity of the limbs, convulsions, and gnathospasmus, accompanied by systemic cyanosis, during the night of December 14. The patient was transferred to the intensive care unit with a PLT count of 71 × 10^9^/L, white cell count of 2.77 × 10^9^/L, neutrophil-granulocyte count of 1.33 × 10^9^/L, and lymphocyte count of 1.34 × 10^9^/L (Table 1). The patient died on the afternoon of December 15 from septic shock, respiratory failure, lactic acidosis, and SFTSV infection involving CNS disturbance.

**Table 1:** Daily laboratory parameters during the whole hospitalization period before the patient died

Date	Platelets (× 10^9^)	White blood cell (× 10^9^)	Neutrophile granulocyte (× 10^9^)	Lymphocyte (× 10^9^)	Lactate dehydrogenase (U/L)	Creatine kinase (U/L)	Activated partial thromboplastin time (s)	Aspartate aminotransferase (U/L)	Alanine aminotransferase (U/L)
December 11	82	1.90	1.22	0.53	NA	NA	NA	NA	NA
December 12	76	1.13	0.81	0.30	466	580	45.6	137	54
December 13	82	2.02	1.56	0.36	1332	2936	NA	427	120
December 14	71	2.77	1.33	1.34	NA	NA	77.9	942	251
December 15	43	1.87	1.22	0.62	3073	9771	164.9	2727	806

*NA* not available

An epidemiological investigation revealed that the patient had visited a small hill 5 km from his home to collect herbs and pick persimmonson December 1 with other people from the same village. From his personal statement, there was no clear history of tick exposure. However, according to his companions, several of them had suffered tick bites when they were hunting and farming on previous occasions. The tick sample collected from the hill was negative for SFTSV nucleic acids. The patient also raised nine chickens, and there were two dogs in his neighborhood. No goats or cows were found in the village. No contacts with a similar syndrome were identified.

**Fig. 1:**
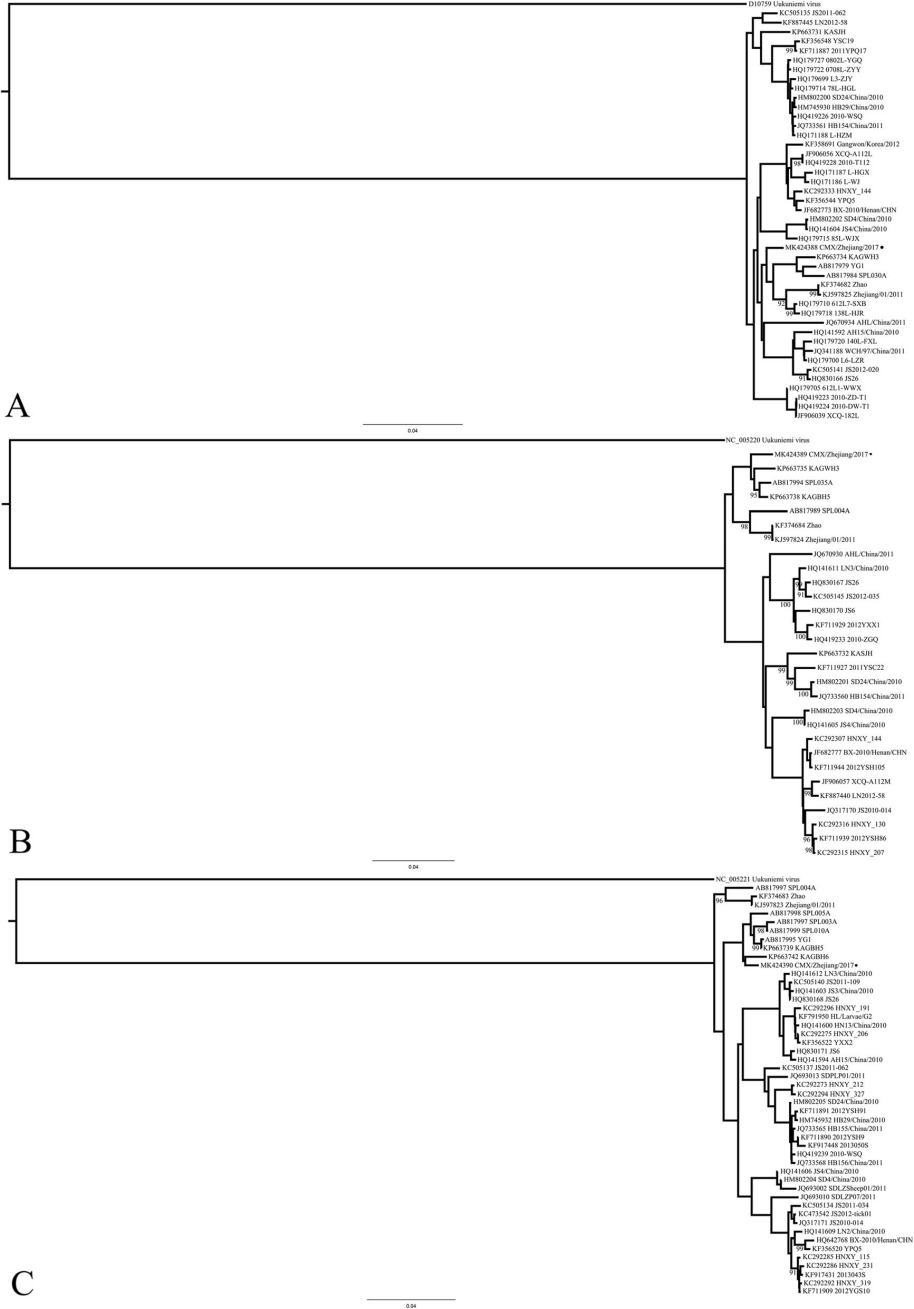
Maximum likelihood trees of sequences of the virus isolated from the patient. Bootstrap values above 90 are indicated at the corresponding nodes. Segments from isolate CMX/2017/Zhejiang are highlighted with circles on each tree. **a** L segment; **b** M segment; **c** S segment

A real-time RT-PCR analysis of a serum sample from the patient indicated that the patient was positive for SFTSV nucleic acids, with a cycle threshold (CT) value of 26. Samples from his contacts and the tick were negative. We obtained the partial gene sequences of the S (1,395 bp), M (2,940 bp), and L (5,245 bp) segments of this SFTSV isolate. A sequence analysis of SFTSV showed 95.8–96.5% sequence identity with known SFTSV isolates at the nucleotide level [10, 15]. This SFTSV isolate had arginine at position 624 in the M glycoprotein segment, which may play a critical role in low-pH-dependent cell fusion activity [16]. An extensive phylogenetic analysis demonstrated that the isolate was closely related to stains from South Korea (KAGWH3/ KAGWH6) (Fig. 1). No intragenic recombination event or segmental reassortment between lineages was detected in the isolate.

## Discussion and conclusions

Here, we have reported a patient with SFTSV infection accompanied by CNS disturbance in Dongyang, Zhejiang Province, China, in 2017. Previous studies have shown that bunyaviruses, such as the Rift Valley fever virus, may be neurotropic [10, 17]. SFTSV-associated CNS disturbance has been observed, but little information is available on the clinical manifestations, epidemiology, and characteristics of the virus [6, 18]. A previously study reported the occurrence of encephalitis in 19.1% of hospitalized SFTS patients, with fatal outcomes in 44.7% of these patients, based on a large sample collected over a long period [6]. In this study, our patient was confirmed as SFTSV infected, and developed clinically diagnosed encephalitis during hospitalization. The diagnosis was based on its sudden onset, and symptoms such as fever, headache, vomiting, and disordered consciousness. The deterioration of consciousness during the course of SFTS is consistently identified as a major risk factor predicting a fatal outcome [5, 7]. We failed to obtain cerebrospinal fluid (CSF) from this patient because his SFTS progressed so rapidly, although other reports have suggested that the observed disturbance of consciousness is an indirect effect of the cytokine storm triggered by SFTSV infection [19]. Screening for SFTSV in patients with encephalitis of unknown cause should be considered, together with screening for common agents that cause viral encephalitis, such as herpes simplex virus type 1.

This SFTSV-infected patient was characterized by the abrupt onset of his fever, together with respiratory tract and gastrointestinal symptoms, followed by a progressive disease course until his death. Laboratory tests showed decreasing trends in his PLT, WBC, neutrophil-granulocyte, and lymphocyte counts, although they were all slightly elevated after treatment on December 13. We monitored the patient’s elevated serum levels of ALT, AST, LDH, and CK, and his elongated aPTT during his hospitalization. These abnormal laboratory parameters were indicative of the pathological lesions in multiple organs and the altered homeostasis of the coagulation system in this patient [1]. Viral replication and the host immune responses are considered to affect the severity and clinical outcome of SFTS [6, 20]. Longer aPTT (> 62.6 s), higher AST (> 288 U/L), or higher blood viral RNA load (> 10^5^) have been suggested to predict a fatal outcome [21, 22]. Reduced consciousness and abnormal laboratory parameters have high predictive value in identifying patients at greater risk of death. In clinical practice, these indicators could be used to reduce the fatality rate and lessen the extent of CNS injury in survivors.

Previous studies based on the extensive phylogenetic analyses have shown that Zhejiang is one of the major areas to import SFTSV from other parts of China and South Korea along migration pathways [11, 15]. The phylogenetic trees we constructed in this study demonstrated that the isolate analyzed originated in South Korea, and differed from isolates derived from mainland of China, including in Shandong, Jiangsu, and Anhui Provinces [23]. SFTS is prevalent in Zhejiang Province, especially between May and August among elderly people living in hilly areas [4]. Our previous seroprevalence study showed that the overall seroprevalence of SFTSV was 7.2% among 986 healthy individuals who reported no symptoms associated with SFTS in the Pujiang district, which is less than 60 km from Dongyang in Zhejiang Province [24]. Other serum surveillance studies have shown that 1.0–3.8% of the examined populations in hilly regions had SFTSV antibodies, which suggests that SFTSV has circulated widely in China [22]. Although this patient denied any clear history of tick exposure, the epidemiological information indicated that the environment and its ecology are typical of those associated with SFTSV [4]. Because CNS involvement in the clinical syndrome predicts a fatal outcome, suspected cases of SFTSV infection in the local area must be monitored closely.

We have reported a patient with SFTSV infection and CNS disturbance in Dongyang, Zhejiang Province, China, in 2017. We have described the clinical, epidemiological, and pathogenic features of this case. The limitations of the study were that: 1) we failed to obtain a CSF sample from the patient because his SFTS progressed rapidly; 2) we collected no whole-genome information for this SFTSV isolate, which should be rectified in a future study. The long-term surveillance of cases of suspected SFTS should be undertaken in SFTSV endemic regions.

## Data Availability

The viral sequences from this patient (CMX/Zhejiang/2017) were deposited in GenBank (MK424388–MK424390). https://www.ncbi.nlm.nih.gov/

## Abbreviations

ALT: Alanine aminotransferase
aPTT: Activated partial thromboplastin time
AST: Aspartate aminotransferase
CDC: Center for Disease Control and Prevention
CK: Creatine kinase
CNS: Central nervous system
CSF: Cerebrospinal fluid
CT: Cycle threshold
LDH: Lactate dehydrogenase
ML: Maximum likelihood
PLT: Platelet
SFTS: Severe fever with thrombocytopenia syndrome
SFTSV: *Severe fever with thrombocytopenia syndrome virus*
WBC: white blood cell
